# Detection of Antibiotic-Resistance Genes in Drinking Water: A Study at a University in the Peruvian Amazon

**DOI:** 10.3390/ijerph22030353

**Published:** 2025-02-27

**Authors:** Euclides Ticona Chayña, Pompeyo Ferro, Eli Morales-Rojas, Ana Lucia Ferro-Gonzales, Jorge Bautista Nuñez, Edwaldo Villanueva Pedraza, Jorge Antonio Malca Florindes, Polan Ferro-Gonzales

**Affiliations:** 1 Facultad de Ciencias Naturales y Aplicadas, Universidad Nacional Intercultural Fabiola Salazar Leguía de Bagua, Jr. Ancash Nro. 520, Bagua 01721, Peru; fferro@unibagua.edu.pe; 2 Instituto de Investigación en Tecnologías de Información y Comunicación (IITIC), Facultad de Ingeniería y Sistemas y Mecánica Eléctrica, Universidad Nacional Toribio Rodríguez de Mendoza de Amazonas, Jr. Libertad Nro 1300, Bagua 01721, Peru; eli.morales@untrm.edu.pe; 3 Departamento Académico de Gestión y Ciencias Sociales, Universidad Nacional de Juliaca, Av. Nueva Zelandia 631, Juliaca 21101, Peru; al.ferrog@unaj.edu.pe; 4 Facultad de Ingenierías, Universidad Nacional Intercultural Fabiola Salazar Leguía de Bagua, Jr. Ancash Nro. 520, Bagua 01721, Peru; jbautista@unibagua.edu.pe; 5 Dirección de Innovación y Transferencia Tecnológica, Universidad Nacional Intercultural Fabiola Salazar Leguía de Bagua, Jr. Ancash Nro. 520, Bagua 01721, Peru; evillanueva@unibagua.edu.pe; 6 Facultad de Ciencias Sociales y Empresariales, Universidad Nacional Intercultural Fabiola Salazar Leguía de Bagua, Jr. Ancash Nro. 520, Bagua 01721, Peru; malcaflorindes@unibagua.edu.pe; 7 Departamento Académico de la Facultad de Ingeniería Económica, Universidad Nacional del Altiplano, Av. Floral No 1153, Puno 21101, Peru; polanf@unap.edu.pe

**Keywords:** antibiotic resistance, antibiotic-resistance genes, conventional PCR, water quality, public health

## Abstract

This study investigated the presence of antibiotic-resistance genes in drinking water consumed by the university community in the Peruvian Amazon. Water samples were collected from three primary sources: inflow from the distribution network, a storage cistern, and an underground intake. Conventional PCR was employed to detect genes associated with resistance to erythromycin (*ermC*), ampicillin (*amp*), ciprofloxacin (*QEP*), multidrug resistance (*marA*), and specific multidrug resistance in *E. coli* (*qEmarA*). Physicochemical analysis revealed compliance with most regulatory standards; however, groundwater samples showed lead concentrations exceeding legal limits (0.72 mg/L) and lacked residual chlorine. All sampling points tested positive for the evaluated resistance genes, demonstrating the widespread dissemination of resistance factors in drinking water. Contrary to initial expectations, resistance genes were also prevalent in treated sources. These findings reveal a critical public health risk for the university community, emphasising the need for effective disinfection systems and robust monitoring protocols to ensure water safety. The presence of these resistance genes in water is a critical public health concern as it can facilitate the spread of resistant bacteria, reducing the effectiveness of medical treatments and increasing the risk of infections that are difficult to control.

## 1. Introduction

Water is essential for human existence. As a finite and increasingly scarce resource, its quality and management for human consumption represent significant global challenges. Each year, nearly two million people worldwide—primarily children under five years old—succumb to waterborne diseases [1]. Although indispensable for life, water quality is severely compromised by emerging contaminants, including antibiotic-resistance genes (ARGs). These genes, found in aquatic environments, pose a critical challenge to global public health due to their ability to transfer horizontally between pathogenic and non-pathogenic bacteria, thereby exacerbating the antimicrobial-resistance crisis [2]. According to the World Health Organisation (WHO), approximately 1.2 million people die annually from antimicrobial-resistant infections, a figure projected to reach 10 million deaths per year by 2050 without effective intervention (WHO, 2021) [3].

Bacteria present in natural habitats have already been identified as potential sources of ARGs. For example, the native soil microbiota has been recognised as a significant reservoir of resistance determinants capable of mobilising within microbial communities [4]. Soil and freshwater environments—including aquifers, groundwater, lakes, rivers, and wastewater—host the most diverse bacterial lineages [5]. Despite this, knowledge about ARGs remains limited. Drinking water derived from natural sources has a high probability of harboring bacteria and hosting ARGs [6]. Recent studies emphasise that water, whether natural or treated, not only acts as a reservoir of ARGs but also facilitates their dissemination through supply systems intended for human consumption [7,8]. This issue is particularly acute in developing countries, where inadequate water treatment and monitoring systems increase the population’s exposure to resistant bacteria, elevating the risk of diseases such as diarrhea, urinary tract infections, and pneumonia caused by multi-resistant bacteria [9,10].

Water acts as a vehicle for disease transmission [11], facilitating the spread of microorganisms [12,13,14] and antibiotic-resistance factors [15,16,17,18]. However, the amplification of resistance genes, especially those of human interest, varies according to the environment, being more frequent in nutrient-rich wastewater and biofilms than in drinking or free-flowing water [19]. As a microbial habitat, water functions as a reservoir and amplifier of resistance genes, facilitating their exchange between pathogenic and non-pathogenic bacteria [12,20,21,22]. Antibiotics not only act as selective agents but also accelerate the evolution of resistance [23]. The remarkable plasticity of ARGs enables them to acquire new or enhanced functionalities beyond their original scope [24].

Despite extensive research, the circumstances under which bacteria in water serve as a significant source of new resistance mechanisms or act as propagators of antibiotic resistance remain unclear [6]. A deeper understanding of bacterial diversity and ecology in aquatic habitats is essential for addressing this issue. Although antibiotics are indispensable in treating infectious diseases in both human and veterinary medicine [14], the indiscriminate disposal of antibiotics into surface, ground, and marine ecosystems aggravates the spread of ARGs. While previous studies suggested that high levels of ARGs result from selective pressure caused by antibiotic contamination [25], recent findings indicate that faecal contamination may play a more significant role [26].

Currently, AGRs are an alarming concern due to their role in the proliferation of antimicrobial resistance, particularly in aquatic environments [2], consequently being a relevant topic of research, including research on both their transport and dissemination (Aminov, 2010). Additionally, the use of organic fertilisers in agricultural fields is a potential pathway for the transmission of antibiotic resistance [11,27,28]. Considering also that animals could serve as potential reservoirs for multi-drug-resistant genes in enterotoxigenic bacteria such as *E. coli* [29], which can spread them through water.

Rainfall can also significantly impact ARGs’ abundance [30]. Moreover, the spread of ARGs is closely associated with the specificity of microorganisms and their interactions [31]. ARGs can disseminate via water bodies and be transferred between bacteria through horizontal gene transfer, thereby intensifying their spread [8].

In Peru, drinking water systems face multiple challenges regarding resource quality and safety. University communities, which often depend on mixed networks of groundwater and treated water, are particularly vulnerable due to inadequate disinfection processes and insufficient monitoring of biological and chemical contaminants. In this regard, the University of Bagua’s water management depends on a mixed system combining the municipal network with underground collection, which increases the risk of cross-contamination. In addition, the lack of constant monitoring and technical training programs for water system operators makes it challenging to guarantee the microbiological and chemical quality of the resource. The absence of regular disinfection, as observed in this study, reflects a structural failure that puts public health at risk and highlights the need for policies that strengthen equitable access to safe water. Based on the above, this study aimed to evaluate the presence of ARGs in the drinking water of a university community in the Peruvian Amazon, providing scientific evidence to improve local strategies for controlling and preventing antimicrobial resistance.

## 2. Materials and Methods

### 2.1. Study Delimitation and Sampling

The study was conducted in the Microbial Biotechnology laboratories of the Fabiola Salazar Leguía National Intercultural University of Bagua in the Amazonas Region of Peru (UNIFSLB).

**Figure 1:**
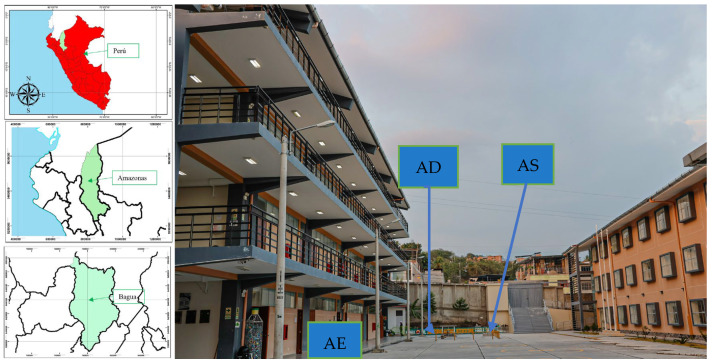
Geographic location of the research.

Sampling was carried out in December 2023 (three repetitions on different dates), collecting samples (duly geo-referenced) from three points of the UNIFSLB according to Figure 1 and Table 1. These sampling points are located in the central campus of the university, characterised by a mean annual accumulated rainfall of 1832 mm, with an annual air temperature of 30 °C, characterised by a very warm climate [32].

Sample collection was carried out following strict protocols, under the criteria established by the Ministry of Health [33,34], ensuring the use of sterile 1 L glass bottles, previously autoclaved and transported under controlled temperature conditions to avoid cross-contamination. One bottle was used for each sampling point, and samples were collected within 15 cm in depth in the case of the cistern, the inlet pipe of the network, and the pipe from the groundwater intake. This followed the protocol of rinsing each bottle three times with the water to be sampled at each scheduled point, then sealing it and placing it in a cooler with freezing gel to preserve it and avoid the incidence of solar radiation until transport to the laboratory. One liter of the sampled water was filtered using cellulose ester filters with a porosity of 0.45 µm and 47 mm in diameter. This process was carried out in the UNIFSLB laboratories.

### 2.2. Analysis of Physical–Chemical Parameters and Heavy Metals

All samples were analysed for mandatory control parameters according to Peruvian regulations [33]: physical–chemical (turbidity, residual chlorine, temperature, conductivity, and pH) and heavy metals (Ni, Pb, and Cr). The legal limits accepted in water for human consumption are indicated in Table 2. The physical–chemical parameters were measured at the point of collection, while heavy metals were measured in the laboratory, no later than 4 h after collection.

The following equipment was used to determine the physical–chemical parameters:

- The HI9828 (Hanna, Smithfield, RI, USA) multiparameter for pH, conductivity and temperature.
- The TN-100 (EUTECH, Aachen, Germany) portable turbidimeter for measuring turbidity.
- Pocket Chlorometer II (Hach, Ames, IA, USA) for residual chlorine with DPD (diethyl-p-phenylenediamine) tablets as reagent.

In the determination of heavy metals, the 4210 MP-AES Atomic Emission Spectrophotometry equipment was used, under method 3120-B, APHA, AWWA, WWF. The Inductively Coupled Plasma (ICP) method for MP-AE was applied to determine nickel, lead, and chromium.

### 2.3. DNA Extraction

The PowerWater^®^ DNA isolation kit (MO BIO Laboratories, Inc., 2746 Loker Ave West, Carlsbad, CA, USA) was used following the manufacturer’s recommended protocol. The quantification of DNA concentration has been performed under the basic photometry procedure using an Eppendorf Fluorescence BioSpectrometer (Hamburg, Germany). Concentration in ng/µL and absorbance conditions (A260) were measured with an optical path of 1 mm. Absorbance at 260 nm (A260) is commonly used to assess DNA purity [35,36].

### 2.4. Determination of Resistance Genes

The determination of resistance genes was carried out by conventional PCR, using the corresponding primers and established conditions (Table 3 and Table 4). In each case, a negative control (ultrapure water instead of sample DNA) was included to detect any contamination by reagents or manipulation. *marA*, *ermC*, *amp*, *QEP* and *qEmarA* were selected due to their local and global relevance. The selection is based on previous studies indicating a high incidence of these genes in water bodies exposed to faecal contamination and urban discharges, which reflects a representative local panorama and contributes to the global context of antimicrobial resistance:

- *marA*: Indicator of multiple antibiotic resistance in *Escherichia coli*, frequent in contaminated aquatic environments [37].
- *ermC*: Encodes resistance to macrolides such as erythromycin, reported in environments related to intensive human activities [8,38].
- *amp*: Associated with resistance to ampicillin, an antibiotic commonly used in human and veterinary medicine [39].
- *QEP*: Representative of resistance to quinolones such as ciprofloxacin, identified in water systems as a key reservoir [18,40].
- *qEmarA*: Specific multiple-resistance indicator in *E. coli*, critical for assessing public health risks [7].

**Table 3:** PCR reactions.

Gene	H_2_O	dNTPs	Buffer (NH)-MgCl_2_)	MgCl_2_	PrimerFW	PrimerRW	Taq	Cell DNA	Reaction Volume	Reference
	UM = ul									
*marA*	13	1	2	1	1	1	0.2	1	20	[37]
*ermC*	16.5	0.4	1	3	0.4	0.4	1.25	2	25	[8,38]
*amp*	13	1	0	1	1	1	0.2	1	20	[39]
*QEP*	13	1	2	1	1	1	0.2	1	20	[18,40]
*qEmarA*	13	1	2	1	1	1	0.2	1	20	[7]

**Table 4:** PCR program and primers.

Gene	Express Resistance to	Programme PCR					
		Conditions	Initial Denaturation	Denaturation	Estimated Primer Annealing Temperature	Estimated Primer Annealing Temperature and Extension	Final Extension Step
***marA***	Multi-antibiotic resistance	T = °C	94	94	55	72	72
		Time	2′	10″	20″	25″	10′
		Repetitions		30			
***ermC***	Eritromicin	T = °C	95	95	54	72	72
		Time	3′	30″	30″	30″	4′
		Repetitions		30			
***amp***	Ampicilin	T = °C	94	94	50	72	72
		Time	5′	1′	3′	3′	15′
		Repetitions		30			
***QEP***	Ciprofloxacin	T = °C	94	94	55	72	72
		Time	2′	10″	20″	25″	10′
		Repetitions		30			
***qEmarA***	Multi-antibiotic-resistance *E. coli*	T = °C	94	94	55	72	72
		Time	2′	10″	20″	25″	10′
		Repetitions		30			

### 2.5. Statistical Analysis

The effect of sampling locations on physical–chemical parameters and DNA concentration was compared using one-way parametric ANOVA with Tukey’s post hoc test. All statistical analyses were performed using SPSS software, version 26.0 (IBM SPSS software, Chicago, IL, USA).

## 3. Results

The physical–chemical parameters of pH, temperature, turbidity, and conductivity evaluated are within the permitted limits established by the regulation of water quality for human consumption in Peru [33], with statistically significant differences observed between sampling points (*p* < 0.05), except for turbidity, where differences are not significant. However, the absence of residual chlorine (0 mg/L in all samples) highlights a lack of adequate water disinfection, thereby increasing the risk of bacterial proliferation. In addition, the concentration of lead in the groundwater samples (0.72 mg/L) significantly exceeds the permitted limit (0.10 mg/L), which represents a toxic risk to public health, due to the food trophic chain and its final arrival to humans, potentially causing chronic intoxication in the university population (Table 5).

**Figure 2:**
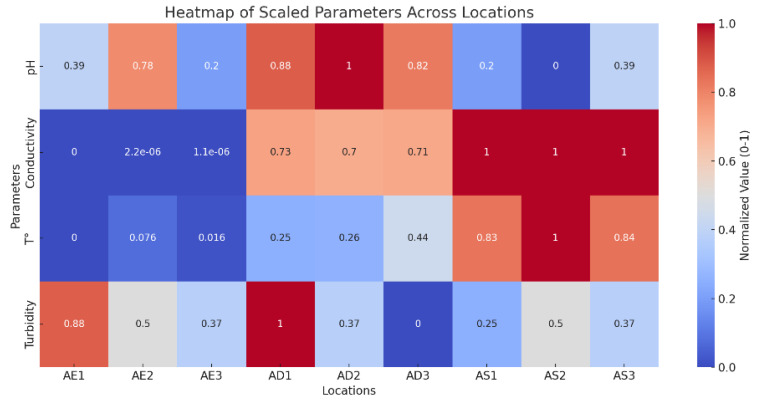
Heat map of physical–chemical parameters.

In the heat map created for the physical–chemical parameters, it is possible to identify locations with high or low values for each parameter. For example, conductivity exhibits its highest values in water of groundwater origin (Figure 2).

The DNA concentrations obtained after DNA extraction are presented in Table 6.

**Figure 3:**
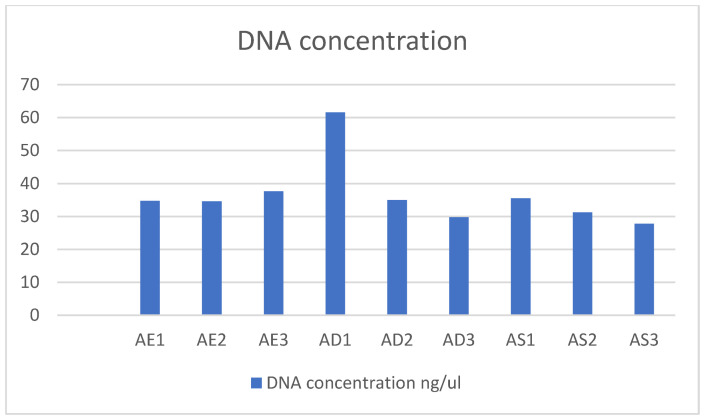
DNA concentration.

DNA concentration varies among samples, from 27.8 ng/µL (AS3) to 61.6 ng/µL (AD1), with no statistically significant differences between sample points (*p* < 0.05). Generally, water samples from the university’s cistern appear to have higher concentrations. While variability exists in DNA concentrations and absorbance conditions, these results enable the performance of PCRs to determine antibiotic-resistance genes (Figure 3).

The resistance genes identified are shown in Table 7. All studied genes (*marA*, *ermC*, *amp*, *QEP* and *qEmarA*) were detected in every sample analysed, evidencing a widespread dissemination of resistance factors in drinking water consumed by the university population. These findings exceed initial expectations, which anticipated lower incidences of resistance genes in samples from treated network systems (redwater entry to UNIFSLB).

## 4. Discussion

Regarding the reported heavy metals, only lead (Pb) exceeded the permitted limit established by Peruvian regulations [33], putting public health at potential risk. This is likely attributable to the metallic structures that contain the water catchment systems and local anthropogenic activities [7,41]. However, Ferro et al. (2024) reported the presence of metals in water for human consumption in Bagua, Peru, coincidentally [42].

The absence of residual chlorine draws attention, as its absence indicates a potential risk of microbial contamination in water consumption [33,43,44]. This is relevant to the university user population, showing evidence of a lack of a disinfection system, since the water captured from the distribution network is combined with groundwater. Therefore, the implementation of a disinfection system for water intended for human consumption is urgently needed, as mandated by the respective authorities [33]. However, in less developed populations, the presence of water without any form of disinfection is common [45,46,47,48]. This obviously poses risks to public health.

Our results indicate that the drinking water of the university population at the Fabiola Salazar Leguía National Intercultural University of Bagua does not meet the requirements for drinking water and suitability for human consumption [33], mainly due to the lack of disinfection. Nevertheless, other physical–chemical parameters comply with Peruvian legislation, with significant differences between the sampled points.

The results of this study, which show a 100% prevalence of antibiotic-resistance genes (ARGs) in the analysed samples, reflect a problem that aligns with global trends but takes on particular characteristics in the local context of the Peruvian Amazon and, specifically, the university population. Studies such as those by Grenni (2022) and Martinez (2009) have reported the presence of ARG in water systems highly influenced by human activities, especially in urban and agricultural areas. However, developing regions such as Bagua face additional challenges due to infrastructure limitations, weak regulations, and insufficient economic resources for efficient water management [7,38].

The DNA concentrations obtained allow us to identify the resistance genes (considering that there are no significant differences between each sampled point), with results consistent with previous research such as Czekalski et al. (2015) [49], who reported the presence of ARGs in water systems contaminated by intensive human activities, and Su et al. (2020) [8], who identified similar resistance genes in water bodies in Asia affected by urban and agricultural discharges. In both cases, insufficient treatment systems were key factors in the persistence of these genes. These results, therefore, contribute to improving the understanding of the origin and spread of resistance genes in aquatic environments. As noted by Nji et al. (2021) [50], water intended for human consumption can act as one of the main reservoirs for the spread of antibiotic resistance through mobile genetic elements [7], particularly in rivers and subway lakes affected by faecal contamination due to a lack of basic sanitation and containing bacteria resistant to antibiotics [51,52,53].

In the case of Bagua, the lack of water chlorination and the mixing of an underground source with the municipal network probably contributed to the spread of ARGs. Local agricultural activities, such as the use of fertilisers and contaminated manure, as well as the dumping of untreated wastewater, are possible additional sources of contamination—a pattern observed in other developing countries [26,54].

The presence of *ermC* (which expresses resistance to macrolides such as erythromycin) and amp (which expresses resistance to ampicillin) indicates a high risk of spreading that is difficult to treat. These findings are especially concerning in communities where antibiotics are used indiscriminately in humans and animals, exacerbating the selection of resistance [2].

The detection of *QEP* and *marA*, which confer resistance to ciprofloxacin and other broad-spectrum antibiotics, represents a critical problem due to the importance of these drugs in modern medicine. Studies such as Janecko et al. (2016) have highlighted the relevance of these resistances in aquatic environments for therapeutic failure in serious infections [55].

Reports of resistance to ampicillin and ciprofloxacin have already been documented [56,57,58]. However, our research also reveals that resistance to these antibiotics is more common than expected. Of course, it is important to consider that these findings pertain to different environments. We agree with Lyimo et al. (2016) [59] that these water sources can be a contamination point for people and animals. Since livestock and humans often share access to water sources, they are likely exposed daily to bacteria with antibiotic-resistance factors, particularly in communities where water treatment options are limited. Therefore, further studies are required to identify the sources of these contaminants and design effective intervention strategies [59].

This implies the need to control antibiotics and their resistance genes present in the environment, as well as address issues in both human and veterinary medicine. This is a first step towards a meaningful contribution to protecting ecosystems and the health of humans and animals [7,54,60,61]. Given the potential transmission of antibiotic-resistant strains through water sources, these findings underscore the importance of ongoing research and increased surveillance to monitor risks. This includes elucidating transmission mechanisms and assessing their impact, consequently identifying and implementing effective interventions [10].

Ciprofloxacin is a critical antibiotic for treating infections, and its use is widespread in both veterinary and human medicine [55]. The detection of resistance genes to ciprofloxacin in the present research is particularly concerning, as this issue is not only ecotoxicological but also contributes to the global crisis of antibiotic resistance and the therapeutic failure associated with its use [55].

It is noteworthy that ampicillin resistance (genes reported in this study) has been found to be transferable in water intended for human consumption, representing a significant risk to public health [62]. Similarly, our findings of genes expressing resistance to erythromycin, which were previously reported, are closely associated with human anthropogenic activity and are thus likely present in aquatic environments for human consumption in our environment [8].

The *qEmarA* gene, which expresses resistance to multiple antibiotics and is specific to *E. coli*, indicates a strong likelihood of *E. coli* presence in the studied drinking water. This suggests the possibility of horizontal gene transfer between commensal and pathogenic bacteria, amplifying the health and ecological impact of water pollution [7].

Our findings are concerning, as they likely correspond to common commensal bacteria in animals and humans. Additionally, these resistance factors may facilitate the transfer of resistance genes within bacterial populations, further complicating the challenge of antimicrobial resistance [63].

Agricultural activities such as the use of contaminated manure and fertilisers may serve as significant sources of resistant bacteria. This pattern has been identified in studies such as Su et al. (2020) [8], where agricultural activities increase the ARG load in water bodies. Furthermore, the lack of adequate treatment, the absence of chlorination systems, and the use of metallic capture systems that release contaminants all contribute to persistent water contamination, aligning with Grenni’s (2022) [7] findings. Similarly, the absence of proper treatment plants for urban wastewater likely plays a central role in the spread of ARGs, as observed in similar contexts across Latin America [54].

The resistance levels detected in this study are comparable to those reported by Su et al. (2020) in Asia and [8] in Europe. Both studies emphasised the importance of constant monitoring and implementing advanced water treatment technologies as critical measures to mitigate the spread of ARGs. However, the Peruvian case highlights a particular challenge due to the combination of local factors, such as insufficient infrastructure and the lack of strict regulations on the use of antibiotics in agriculture and livestock.

In Europe, Grenni (2022) [7] demonstrated how advanced treatment technologies and a strict regulatory framework have effectively mitigated the spread of ARGs in water systems. In contrast, in Bagua, the lack of adequate disinfection (evidenced by the absence of residual chlorine) and lead contamination in groundwater reflect critical deficiencies in water management. This underlines the importance of adapting globally proven solutions to local realities, considering existing infrastructure and socioeconomic conditions.

Although local water management policies in Peru are governed by the Water Quality Regulation for Human Consumption [33], which establishes clear parameters for water quality, their application in rural and peri-urban areas is inconsistent due to a lack of resources and technical capacities.

In view of this, the implementation of UV-C disinfection systems and membrane filtration could be an effective solution to eliminate both bacteria and resistance genes. These technologies have proven effective in global studies [8]. Additionally, granular activated carbon or ozonation processes could complement these technologies to remove chemical contaminants, such as the detected heavy metals.

Furthermore, establishing systematic monitoring programs to assess water quality and the prevalence of ARGs is essential to detect risks in a timely manner. These initiatives may include using technologies such as qPCR for more sensitive and specific detection.

It is essential to invest in the training of technical staff and infrastructure at the university to ensure compliance with water quality standards. This includes the development of automated chlorination systems and laboratories capable of performing advanced microbiological analyses.

At a global level, the findings of this study align with patterns observed in Asia and Africa, where water systems frequently lack adequate treatment [53]. However, the prevalence of ARGs in the studied water suggests a significant influence of local factors, such as faecal contamination and limited sanitation infrastructure, which should be addressed in future research.

Furthermore, the cumulative impact of these resistances on human health needs to be assessed, considering not only direct exposure to contaminated water but also indirect routes. This may require additional phenotypic and genotypic studies to characterise the bacteria present and better understand their resistance dynamics. Likewise, future research should take into account more sampling points over a longer period of time. This could not be carried out in the study presented, due to the low economic resources of the project to obtain inputs and reagents for the analysis of physicochemical, genomic, and metagenomic parameters that would allow a more specialised characterisation.

## 5. Conclusions

The results of this study have demonstrated the presence of antibiotic-resistance genes (ARGs) in all water samples analysed at the Universidad Nacional Intercultural Fabiola Salazar Leguía de Bagua, with a prevalence of genes such as *ermC*, *amp*, *QEP*, *marA* and *qEmarA*. These findings indicate a significant risk to public health, especially in a vulnerable population such as the university, since it is an intercultural institution and houses students with very low economic resources.

It is essential to propose the implementation of a constant monitoring program for the detection of ARGs in water sources. Using technologies such as qPCR for the detection of specific genes could provide faster and more accurate results, allowing timely interventions.

Promoting restrictions on the use of antibiotics in agricultural activities and establishing policies that limit the indiscriminate use of antibiotics, especially in areas close to sources of drinking water, through the regulation of antibiotics use and the promotion of more sustainable practices are essential to reduce the selective pressure that facilitates the spread of ARGs.

Additional research studies should be conducted to isolate and phenotypically and genetically characterise resistant bacteria present in water. This would provide valuable information to understand the diversity of ARGs in the region and develop more targeted intervention strategies.

Simultaneous evaluation of the presence of chemical and microbiological contaminants in water, such as heavy metals (e.g., lead) and ARGs, is necessary to design more comprehensive solutions that address both types of contamination.

Antibiotic resistance in aquatic environments is a complex problem that affects not only humans but also animals and the environment, making it imperative to adopt a One Health approach. Coordinating efforts between human health, veterinary, and environmental sectors is essential to comprehensively address the spread of antimicrobial resistance (AMR) through water.

This study highlights the urgent need to adopt practical and sustainable measures to improve water quality and reduce the spread of antimicrobial resistance in the region. The implementation of advanced treatment technologies, such as membranes and UV-C, along with stricter policies on antibiotic use and constant monitoring of water quality, represents critical steps to protect public health and mitigate the impact of AMR on the aquatic environment.

Subsequent research should be carried out in the same sampling locations, and based on our results, *E. coli* should be isolated in order to characterise it phenotypically and genotypically. Based on our results, genomic and metagenomic studies should be promoted to allow a more specialised characterisation of antimicrobial resistance at the university and in the Amazonas Region.

## Figures and Tables

**Table 1:** Sampling location at the university.

Samples	Location	Georeference			Observations
		East	North	Altitude	
AE1	Redwater entry to UNIFSLB	773167	9376966	485	Redwater EMAPAB *
AE2					
AE3					
AD1	UNIFSLB Cistern	773166	9376965	485	Redwater mixture with underground water
AD2					
AD3					
AS1	UNIFSLB water collection	773166	9376965	485	Underground water capture
AS2					
AS3					

* Municipal Drinking Water and Sewerage Bagua.

**Table 2:** Evaluated parameters.

Parameter	Legal Limit
	(DS N° 031-2010-SA, MINSA 2010)
**Physical–chemical**	
turbidity	5 NTU
residual chlorine	0.5 mg Cl^−^/L
temperature	na
conductivity (25 °C)	1500 μhmo/cm
pH	6.5–8.5
**Heavy metals**	
Nickel, Ni	0.02 mg/L
Lead, Pb	0.10 mg/L
Chrome, Cr	0.050 mg/L

**Table 5:** Physicochemical parameters and heavy metals.

Parameters	UM	AE1	AE2	AE3	AD1	AD2	AD3	AS1	AS2	AS3
pH **	pH unit	6.9	7.1	6.8	7.15	7.21	7.12	6.8	6.7	6.9
Conductivity **	μmHmo/cm	0.049	0.051	0.05	657	634	645	903	901	904
T^0^ **	C^0^	25.69	26.02	25.76	26.77	26.8	27.6	29.29	30.01	29.31
**Residual chlorine**	mgL^−1^	0	0	0	0	0	0	0	0	0
Turbidity *	NTU	1.6	1.3	1.2	1.7	1.2	0.9	1.1	1.3	1.2
**Heavy metals**										
**Nickel, Ni**	mg/L	0	0	0	0	0	0	0	0	0
**Lead, Pb**	mg/L	0	0	0	0	0	0	0.72	0.72	0.72
**Chrome, Cr**	mg/L	0	0	0	0	0	0	0	0	0

** Statistically differences. * Not statistically significant.

**Table 6:** DNA concentration.

Sampling	DNA * Concentration ng/µL	Conditions A260/1 mm
AE1	34.7	0.095
AE2	34.6	0.117
AE3	37.6	0.0999
AD1	61.6	0.167
AD2	35	0.112
AD3	29.7	0.077
AS1	35.5	0.102
AS2	31.2	0.077
AS3	27.8	1.786

* Not statistically significant.

**Table 7:** Resistance genes.

Sampling	Location	Resistance Genes				
		*marA*	*ermC*	*amp*	*QEP*	*qEmarA*
AE1	Redwater entry to UNIFSLB	+	+	+	+	+
AE2		+	+	+	+	+
AE3		+	+	+	+	+
AD1	UNIFSLB Cistern	+	+	+	+	+
AD2		+	+	+	+	+
AD3		+	+	+	+	+
AS1	UNIFSLB water collection	+	+	+	+	+
AS2		+	+	+	+	+
AS3		+	+	+	+	+

## Data Availability

All the raw data is available on request.
